# Lichen sclerosus in paediatric patients with phimosis undergoing circumcision

**DOI:** 10.1093/skinhd/vzaf065

**Published:** 2025-10-07

**Authors:** Nada Aboukhatwah, Sandra Jerkovic Gulin, Christopher B Bunker, Michalis Varnavas, Georgios Kravvas

**Affiliations:** Department of Dermatology, Whittington Health NHS Foundation Trust, London, UK; Department of Dermatology and Venereology, Ryhov County Hospital, Jönköping, Sweden; Division of Cell Biology, Department of Biomedical and Clinical Sciences, The Faculty of Medicine and Health Sciences, Linköping University, Linköping, Sweden; Department of Dermatology, University College London Hospitals NHS Foundation Trust, London, UK; Department of Urology, Whittington Health NHS Foundation Trust, London, UK; Department of Dermatology, Whittington Health NHS Foundation Trust, London, UK; Department of Dermatology, University College London Hospitals NHS Foundation Trust, London, UK

## Abstract

**Background:**

Male genital lichen sclerosus is a relatively uncommon but increasingly recognized cause of phimosis in paediatric patients.

**Objectives:**

To investigate the presence of male genital lichen sclerosus (MGLSc) in paediatric patients presenting with phimosis and to evaluate the diagnostic accuracy of clinical assessment compared with histopathological findings.

**Methods:**

We conducted a single-centre, retrospective study of 48 paediatric circumcisions performed for phimosis over an 18-month period. Clinical diagnoses of phimosis were categorized as physiological or pathological and compared with postoperative histopathological findings. To quantify diagnostic reliability, percentage agreement and Cohen’s kappa coefficient were used.

**Results:**

MGLSc was confirmed histologically in 71% of patients, with the highest proportion occurring in boys aged 9–11 years. Among those clinically diagnosed with physiological phimosis, 70% showed histological evidence of MGLSc, of whom 48% exhibited extensive disease. The overall concordance between clinical and histological diagnoses was 43.75%, with a Kappa value of 0.027, indicating only slight agreement.

**Conclusions:**

Currently, clinical assessments seem to underestimate the proportion of cases with MGLSc, especially among younger boys. Enhancing awareness and recognition of this condition is essential to facilitate accurate and timely diagnosis, and to guide appropriate interventions, thereby preventing disease progression and reducing associated morbidity.

## Teaser

Male genital lichen sclerosus (MGLSc) is a chronic dermatosis and a key factor in paediatric pathological phimosis, yet its presence is often underestimated. In this study, 71% of cases of paediatric circumcision for phimosis showed histological evidence of MGLSc. Alarmingly, 70% of cases clinically diagnosed as physiological phimosis were confirmed as MGLSc, revealing significant discrepancies between clinical and histological diagnosis that warrant a reevaluation of clinical approaches to this condition. These findings emphasize the need for heightened awareness of the presence of this condition to facilitate timely diagnosis and mitigate disease progression.

What is already known about this topic?

- Male genital lichen sclerosus is a relatively uncommon but increasingly recognized cause of phimosis in paediatric patients, as highlighted in emerging literature.

What does this study add?

- This study contributes to the growing evidence suggesting that the presence of male genital lichen sclerosus among paediatric patients with phimosis is significantly underestimated, and evaluates the diagnostic concordance between clinical and histological assessments.

Phimosis, the inability to retract the prepuce over the glans penis, is a common presentation in paediatric and adult medicine. In the paediatric context, phimosis is generally categorized as either physiological or pathological. Physiological phimosis represents a normal developmental stage in male infants, typically resolving spontaneously as the child ages and the preputial tissues become more pliable.^1^ Conversely, pathological phimosis results from acquired fibrosis of the distal foreskin, often secondary to chronic inflammation, recurrent infections or mechanical trauma.^2,3^

Male genital lichen sclerosus (MGLSc) is a chronic inflammatory dermatosis and a well-established aetiological factor in pathological phimosis across all age groups.^3^ Contrary to earlier assumptions of rarity, recent population studies demonstrate a notable incidence of lichen sclerosus (LS), suggesting it is more common than previously thought and highlighting the need for increased clinical vigilance.^4^

This study aimed to quantify the proportion of cases with MGLSc in paediatric patients undergoing circumcision for phimosis, with the objective of clarifying its role in pathological paediatric phimosis and enhancing diagnostic accuracy to support timely intervention.

## Patients and methods

We conducted a single-centre retrospective review of paediatric circumcisions performed in our urology department (Whittington Health NHS Foundation Trust, London, UK) over an 18-month period. The patient cohort consisted of 48 patients, ranging in age from 1 to 17 years, who underwent circumcision for phimosis between January 2023 and June 2024. Patients aged 18 years and older, as well as those undergoing circumcision for social or cultural reasons, were excluded.

Data collection involved review of clinic letters, pre- and postoperative notes, and histopathology reports. The clinical indications for circumcision and histological results were analysed and categorized.

Clinical and histological examination of all patients was performed at a specialist centre by experienced consultant urologists and histopathologists. Clinical diagnosis of MGLSc was based on a history of phimosis affecting a previously retractable prepuce, or a nonstretchy prepuce with features of sclerosis, including whitening, thickening, a scar-like appearance, fibrotic rings or a sclerotic appearance of the visible glans. Histopathological diagnosis was based on established histological criteria for MGLSc, including features such as epidermal atrophy, oedema and hyalinization of the upper dermis. Foreskin samples were evaluated as whole specimens in all cases.

Diagnostic concordance was assessed by comparing the initial clinical diagnosis to the corresponding histopathological results. Concordance was expressed both as a percentage and through the application of Cohen’s kappa coefficient, a statistical measure used to assess interobserver reliability for qualitative variables. The kappa values are interpreted as follows: <0 as no agreement, 0–0.20 slight agreement, 0.21–0.40 fair agreement, 0.41–0.60 moderate agreement, 0.61–0.80 substantial agreement and 0.81–1 as almost perfect agreement.^5^ This analysis was applied to determine how frequently MGLSc may have been clinically underdiagnosed or misdiagnosed.

## Results

The study cohort included 48 paediatric patients, aged 1–17 years [mean (SD) age 8.9 (4.5) years; median 8 years (range 1–17)]. Of these, 33 patients (69%) were preoperatively diagnosed with physiological phimosis, while 15 (31%) were diagnosed with pathological phimosis, suspected to be secondary to MGLSc.

Among those diagnosed with physiological phimosis, 2 patients (6%) had asymptomatic but persistent phimosis, whereas 31 (94%) reported symptoms, including discomfort, recurrent urinary tract infections or episodes of balanoposthitis (Table 1).

**Table 1:** Correlation between clinical diagnosis and histological findings in paediatric patients undergoing circumcision for phimosis

	Histological diagnosis			
	**Normal (*n*)**	**Nonspecific chronic inflammation (*n*)**	**Lichen sclerosus (*n*)**	**Total (*n*)**
Clinical impression				
Asymptomatic physiological phimosis	0	0	2	2
Symptomatic physiological phimosis	3	7	21	31
Lichen sclerosus	2	2	11	15
Total	5	9	34	48

### Histological findings

Histopathological analysis confirmed MGLSc in 34 of 48 cases (71%), with the highest proportion observed in boys aged 9–11 years (mean age 9.4 years). Of these patients positive for MGLSc, 5 (15%) exhibited ­early-stage changes, 10 (29%) demonstrated focal disease and 19 (56%) showed extensive involvement.

Nonspecific chronic inflammation was found in nine patients (19%), while five (10%) showed normal histology. The mean (SD) age of patients with histologically confirmed MGLSc was higher [9.4 (4.5) years] than those with an unremarkable histology [mean (SD) 5.8 (2.7) years].

### Age and severity correlation

**Figure 1:**
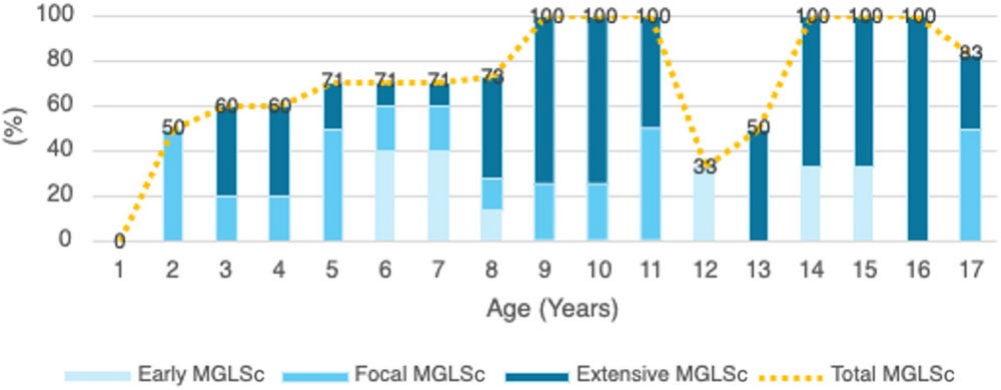
Age-based analysis of the presence and histological extent of male genital lichen sclerosus (MGLSc) in paediatric phimosis. Light blue bars represent early-stage MGLSc, medium blue bars indicate focal MGLSc and dark blue bars denote extensive MGLSc. The yellow dashed line depicts the overall presence of MGLSc.

Statistical analysis demonstrated an age-related increase in the detection and severity of MGLSc. MGLSc was identified in 50% of patients aged 2 years (*n* = 2/4), with the proportion gradually rising to 64% among those aged 3–8 years (*n* = 14/22) and reaching 100% in those aged 9–11 years (*n* = 8/8). A transient decline was observed in the 12–13 years age group (40%, *n* = 2/5), before rising again to an average of 89% in patients aged 14 years and above (*n* = 8/9) (Figure 1).

The severity of MGLSc demonstrated a positive correlation with age. Younger patients predominantly exhibited early or focal disease, whereas extensive involvement became increasingly prevalent in older patients, ranging from 0% in 2-year-old patients to 100% in specific older cohorts (Figure 1).

### Diagnostic concordance

Histology revealed MGLSc in 23 of 33 patients (70%) initially classified as having physiological phimosis, with 48% (*n* = 11) of these showing extensive disease. Nonspecific inflammatory findings were noted in seven patients (21%), while there were no pathological findings in three patients (9%).

Among the 34 patients with histologicall confirmed MGLSc, only 11 (32%) were accurately identified as MGLSc preoperatively. Of the 15 patients operated on with a clinical suspicion of MGLSc, histological confirmation was achieved in 11 (73%), while the remaining 4 showed either nonspecific inflammation (*n* = 2) or normal histology (*n* = 2).

Overall, the diagnostic concordance between clinical and histological findings was 43.75%, with a Cohen’s kappa coefficient of 0.027, indicating only slight agreement between the two diagnostic approaches.

## Discussion

Phimosis, defined as the inability to retract the prepuce over the glans penis, is frequently encountered in paediatric and adult medical practice. In paediatrics, it is typically classified as physiological, when expected to resolve spontaneously with time, or pathological, when arising from acquired causes.^1^ However, the term ‘phimosis’ is sometimes also inaccurately used to describe difficulty in retraction rather than true inability to retract the prepuce.

Physiological phimosis, present in all male infants at birth, results from natural adhesions between the inner foreskin and the glans penis formed during fetal development.^3^ Over time, these adhesions are expected to separate, and the foreskin ought gradually to become retractable, aided by erections and eukeratinization of the inner epithelium.^3^ A large-scale study by Hsieh *et al*. demonstrated that foreskin retractability increases with age, rising from 8.2% in 6-year-olds to 58.1% by age 12 years, while physiological phimosis declines from 17.5% to 1.2% in the same age group, suggesting that the majority of phimoses resolve over time without intervention.^6^

The mechanisms governing this resolution and the precise age range for expected resolution have not yet been fully elucidated. Consequently, diagnosing pathological phimosis can be challenging and often relies on the presence of fibrotic rings, symptoms such as pain and swelling, or urinary difficulties.^7^

The term ‘physiological’ has been traditionally used to describe cases of phimosis expected to resolve naturally; however, this designation may be misleading in cases where underlying pathology, such as MGLSc, is present.

However, the term ‘physiological’ phimosis is inherently retrospective, as it implies a natural resolution that can only be confirmed over time. In real-time clinical practice, this designation may not be appropriate. We therefore propose that phimosis be more accurately classified as either ‘idiopathic’ or ‘secondary to an identifiable cause’ such as MGLSc. This distinction may help improve diagnostic clarity and guide management.

While physiological phimosis is expected to resolve with age, its persistence beyond a certain age, particularly after the age of 7 or 8 years, should raise concern for underlying pathology. As such, confidently diagnosing a case as ­‘physiological’ becomes increasingly uncertain with age, and caution should be exercised in applying this term to older children.

MGLSc is a chronic inflammatory dermatosis and a well-­documented cause of pathological phimosis in paediatric and adult male populations.^7,8^ Symptoms of MGLSc include foreskin tightness, pain, pruritus and fissuring, with variable signs such as sclerosis, lichenoid and zoonoid inflammation, pigmentary changes, architectural effacement and adhesions.^8–11^ Without appropriate treatment, MGLSc can lead to urethral strictures and meatal stenosis, and is a recognized risk factor for penile intraepithelial neoplasia, squamous cell carcinoma and penile melanoma.^12–16^

The disease generally shows a trimodal age distribution, peaking in childhood, and in the fourth and fifth decades of life.^17^ Although the exact aetiology of MGLSc remains disputed, the bulk of the evidence points towards chronic, occluded exposure of susceptible epithelium to urine under the prepuce as the primary cause.^11,18,19^

Consequently, MGLSc typically affects uncircumcised male patients or those who develop a neoforeskin after circumcision.^11^ However, this mechanism does not fully account for cases of extragenital LS where similar occlusive environments are not present. Alternative or complementary mechanisms, such as Koebnerization (the isomorphic response to friction or trauma), genetic susceptibility and autoimmunity, have also been proposed.^20,21^ It is likely that the pathogenesis of LS is multifactorial, with urine exposure playing a key role in susceptible individuals under specific conditions.

Our study underscores the underappreciated presence of MGLSc in a significant percentage of patients with paediatric phimosis. Histopathology confirmed MGLSc in 71% of children undergoing circumcision for phimosis; this is markedly higher than the 31% suspected based on clinical diagnosis alone. This discrepancy aligns with previous findings that MGLSc is often clinically underdiagnosed, especially in younger children.^17,22–24^ The literature suggests an incidence of 1 in 200 boys. However, given the poor clinical recognition of MGLSc and the limitations of histopathological diagnosis, the true prevalence is likely impossible to ascertain.^25^

Historically, MGLSc has been considered to affect primarily school-aged boys.^26^ However, recent studies, including our own, have identified histologically confirmed cases in boys as young as 2 years.^27,28^ Our findings also corroborate previous reports of a peak incidence among boys aged 9–11 years, with the proportion and severity of MGLSc increasing with age.^2,29,30^ The mean age of children with histologically confirmed pathological phimosis was significantly higher than those without pathological findings, supporting a potential progression of disease severity over time.

Our findings indicate a significant discrepancy between clinical and histological diagnoses, with an overall diagnostic concordance of 43.75% (kappa = 0.027). This diagnostic gap highlights the known, existing challenges of identifying MGLSc based on clinical features alone, as it is often mistaken for other causes of phimosis such as physiological adhesions or chronic balanitis.^10^

Importantly, it must be acknowledged that the histological confirmation of MGLSc can also be challenging, particularly in early or partially treated cases where hallmark features, such as epithelial thinning and fibrosis, may not be readily apparent, leading to false-negative results.^31–33^

This may help explain why five of the foreskin samples showed normal histology despite clinical indications for surgery. In these cases, nonspecific adhesions, low-grade inflammation or the effects of prior topical corticosteroids may have contributed to the absence of diagnostic features of MGLSc.

This mechanism may also explain the higher male-to-female incidence ratio in childhood genital LS, and, alongside the circumcisions that take place in childhood, also contribute to the lower male-to-female incidence ratio in adult genital LS. However, this may also be partly due to underdiagnosis and reporting.

Physiological phimosis during early childhood often leads to increased urinary occlusion beneath the prepuce, creating a moist and occlusive environment that may predispose children to the development of MGLSc and subsequent progression to pathological phimosis.^18,34,35^ This mechanism highlights the potential role of urine exposure as a contributing factor in the pathogenesis of MGLSc. It also provides a plausible explanation for the observed higher male-to-­female incidence ratio of genital LS in childhood.

Moreover, the practice of childhood circumcision, which eliminates the occlusive environment of the foreskin, may contribute to the relatively lower male-to-female incidence ratio of genital LS observed in adults.^25,36,37^ By eliminating prolonged exposure of the preputial epithelium to irritants such as urine, circumcision significantly reduces the risk of MGLSc in male patients who undergo the procedure during childhood. However, this protective effect may be diminished in adulthood if conditions such as obesity lead to the development of a pseudoforeskin, recreating an occlusive environment that could predispose to MGLSc over time.^38^ It is also possible that the relatively lower male-to-female incidence ratio of genital LS observed in adults may also be partly due to underdiagnosis and lack of reporting.

Our findings suggest that the diagnostic approach to phimosis in children requires refinement to address the current underdiagnosis. It is important to improve awareness about paediatric MGLSc among primary and secondary care clinicians. Furthermore, clinicians should maintain a high index of suspicion for MGLSc in all paediatric phimoses, particularly in older boys and those with recurrent infections or failed conservative treatments. Persistent or symptomatic phimosis, especially in boys over the age of 7 or 8 years, should be regarded as potentially pathological and considered MGLSc until proven otherwise. This shift in diagnostic mindset may prompt earlier recognition, referral and intervention.

However, relying solely on histological examination for the diagnosis of MGLSc can also be problematic. Diagnostic biopsies are often prone to sampling error, potentially missing affected areas, due to the patchy nature of the disease, and are therefore not routinely recommended. This limitation underscores the importance of a thorough clinical assessment for accurate diagnosis. Additionally, when topical corticosteroids are used as part of conservative management, they may partially or completely resolve the presence of lichenoid inflammation, a key histopathological feature of LS. This effect can further complicate the histological diagnosis, as the characteristic signs of LSc may be masked, leading to false-negative results. Consequently, clinicians must weigh the limitations of histology and incorporate clinical judgement in managing suspected cases of MGLSc.

Currently, routine histopathological analysis of excised foreskin samples from circumcisions is not universally practised.^39,40^ We strongly recommend implementing it as a standard procedure in all cases. Routine histological confirmation of MGLSc not only facilitates accurate diagnosis, but also carries important prognostic and medicolegal implications. It can inform parental counselling, influence decisions regarding long-term monitoring and support early recognition of potential complications such as urethral involvement or recurrence.

A diagnosis of LS has significant implications. Clinicians ought to inform patients and parents about the noxious effects of occluded exposure to urine and advise them to seek medical input should a pseudo- or neoforeskin develop in the future. Furthermore, the high proportion of MGLSc in our cohort should act as a guide for surgeons to perform full circumcision rather than a preputioplasty or partial circumcision in paediatric phimosis. While preputioplasties and partial circumcisions may temporarily alleviate symptoms, they fail to fully address the underlying cause of MGLSc. These procedures leave a portion of the foreskin intact, allowing the occlusive environment to persist and predisposing the patient to the recurrence or progression of MGLSc in the future. However, full circumcision significantly reduces the risk of disease progression and recurrence by eliminating the occlusive environment.

These results also have important implications for clinical care pathways. Persistent or symptomatic phimosis, particularly in older children, should prompt earlier consideration of MGLSc. This could justify expedited referral to dermatology or urology, and, in some cases, early surgical intervention. Incorporating histological assessment into routine practice can also help stratify patients for long-term follow-up, especially where urethral or recurrent disease is a concern.

This diagnostic challenge is not unique to paediatric populations. Evidence from adult cohorts has similarly highlighted the under-recognition of MGLSc, with delayed diagnoses often leading to more advanced disease and complex interventions.^41^ A multidisciplinary approach involving dermatology, urology and pathology has the potential to improve diagnostic accuracy and reduce surgical morbidity.

In clinical practice, we follow a stepwise approach to managing boys with phimosis, in accordance with current European Society for Paediatric Urology guidelines.^42^ Initial treatment typically involves a short course of potent topical corticosteroids (e.g. 0.05% clobetasol propionate ointment applied twice daily for 4 weeks), combined with advice on gentle manual retraction and hygiene. If phimosis persists despite conservative therapy, we recommend proceeding to full circumcision with histological examination of the excised foreskin. This approach aims to balance conservative management with timely surgical intervention, thereby reducing the risk of disease progression and associated complications.

The strengths of this study include the consistent examination of the entire prepuce for histopathological confirmation, which enhances diagnostic accuracy, as well as the systematic and methodical approach to data collection. However, several limitations must be acknowledged. Firstly, the retrospective design may introduce selection bias. Secondly, the study’s findings are limited to a single centre, potentially impacting the generalizability of results. Thirdly, the influence of prior treatments, such as topical corticosteroids, on histopathological findings was not controlled. Future prospective, multicentre studies could address these gaps.

## Data Availability

The data underlying this article will be shared on reasonable request to the corresponding author.

## References

[1] Øster J . Further fate of the foreskin. Incidence of preputial adhesions, phimosis, and smegma among Danish schoolboys. Arch Dis Child 1968; 43:200–3. 5689532 10.1136/adc.43.228.200 PMC2019851

[2] Gargollo PC, Kozakewich HP, Bauer SB et al Balanitis xerotica obliterans in boys. J Urol 2005; 174:1409–12. 16145451 10.1097/01.ju.0000173126.63094.b3

[3] Shahid SK . Phimosis in children. ISRN Urol 2012; 2012:707329. 23002427 10.5402/2012/707329 PMC3329654

[4] Jerkovic Gulin S, Lundin F, Eriksson O, Seifert O. Lichen sclerosus-incidence and comorbidity: a nationwide Swedish register study. J Clin Med 2024; 13:2761. 38792303 10.3390/jcm13102761 PMC11122656

[5] Landis JR, Koch GG. The measurement of observer agreement for categorical data. Biometrics 1977; 33:159–74. 843571

[6] Hsieh TF, Chang CH, Chang SS. Foreskin development before adolescence in 2149 schoolboys. Int J Urol 2006; 13:968–70. 16882064 10.1111/j.1442-2042.2006.01449.x

[7] Fox W, McKenna PH. Treatment algorithm for the comprehensive management of severe lichen sclerosus in boys based on the pathophysiology of the disease. J Pediatr Urol 2024; 20:S66–73. 38918118 10.1016/j.jpurol.2024.06.007

[8] Kravvas G, Shim TN, Doiron PR et al The diagnosis and management of male genital lichen sclerosus: a retrospective review of 301 patients. J Eur Acad Dermatol Venereol 2018; 32:91–5. 28750140 10.1111/jdv.14488

[9] Kantere D, Löwhagen GB, Alvengren G et al The clinical spectrum of lichen sclerosus in male patients – a retrospective study. Acta Derm Venereol 2014; 94:542–6. 24549239 10.2340/00015555-1797

[10] Bunker CB, Shim TN. Male genital lichen sclerosus. Indian J Dermatol 2015; 60:111–17. 25814697 10.4103/0019-5154.152501 PMC4372901

[11] Edmonds EV, Hunt S, Hawkins D et al Clinical parameters in male genital lichen sclerosus: a case series of 329 patients. J Eur Acad Dermatol Venereol 2012; 26:730–7. 21707769 10.1111/j.1468-3083.2011.04155.x

[12] Sim SJY, Dear K, Mastoraki E et al Genital lichen sclerosus and melanoma; a systematic review. Skin Health Dis 2023; 3:e198. 37013116 10.1002/ski2.198 PMC10066758

[13] Dear K, Kravvas G, Sim S et al Primary penile melanoma and genital lichen sclerosus. Skin Health Dis 2023; 3:e274. 38047263 10.1002/ski2.274 PMC10690690

[14] De Luca DA, Papara C, Vorobyev A et al Lichen sclerosus: the 2023 update. Front Med 2023; 10:1106318. 10.3389/fmed.2023.1106318 PMC9978401 36873861

[15] James ML, Kravvas G, Lallas A, Bunker CB. The clinical and dermatoscopic features of penile pigmentation in men with genital lichen sclerosus. Skin Health Dis 2024; 4:e435. 39355751 10.1002/ski2.435 PMC11442078

[16] Kravvas G, Ge L, Ng J et al The management of penile intraepithelial neoplasia (PeIN): clinical and histological features and treatment of 345 patients and a review of the literature. J Dermatol Treat 2022; 33:1047–62. 10.1080/09546634.2020.1800574 32705920

[17] Choudhary C, Beazley R, Uppal E et al The age-related incidence of male genital lichen sclerosus is triphasic. Skin Health Dis 2024; 4:e447. 39624752 10.1002/ski2.447 PMC11608886

[18] Kravvas G, Muneer A, Watchorn RE et al Male genital lichen sclerosus, microincontinence and occlusion: mapping the disease across the prepuce. Clin Exp Dermatol 2022; 47:1124–30. 35150005 10.1111/ced.15127

[19] Panou E, Panagou E, Foley C et al Male genital lichen sclerosus associated with urological interventions and microincontinence: a case series of 21 patients. Clin Exp Dermatol 2022; 47:107–9. 34499360 10.1111/ced.14869

[20] Terlou A, Santegoets LAM, van der Meijden WI et al An autoimmune phenotype in vulvar lichen sclerosus and lichen planus: a Th1 response and high levels of microRNA-155. J Invest Dermatol 2012; 132:658–66. 22113482 10.1038/jid.2011.369

[21] Uthayakumar AK, Kravvas G, Bunker CB. Extragenital lichen sclerosus arising in tattooed skin. BMJ Case Rep 2022; 15:e246216. 10.1136/bcr-2021-246216 PMC8739425 34992057

[22] Jerkovic Gulin S, Liljeberg L, Seifert O. The impact of genital lichen sclerosus in men and women on quality of life: a prospective cohort study. Int J Womens Dermatol 2024; 10:e131. 38240010 10.1097/JW9.0000000000000131 PMC10796136

[23] Gulin SJ, Lundin F, Seifert O. Comorbidity in patients with lichen sclerosus: a retrospective cohort study. Eur J Med Res 2023; 28:338. 37697418 10.1186/s40001-023-01335-9 PMC10494448

[24] Bochove-Overgaauw DM, Gelders W, De Vylder AM. Routine biopsies in pediatric circumcision: (non) sense? J Pediatr Urol 2009; 5:178–80. 19138882 10.1016/j.jpurol.2008.11.008

[25] Kumar KS, Morrel B, van Hees CLM et al Comparison of lichen sclerosus in boys and girls: a systematic literature review of epidemiology, symptoms, genetic background, risk factors, treatment, and prognosis. Pediatr Dermatol 2022; 39:400–8. 35229894 10.1111/pde.14967 PMC9545843

[26] Meuli M, Briner J, Hanimann B, Sacher P. Lichen sclerosus et atrophicus causing phimosis in boys: a prospective study with 5-year followup after complete circumcision. J Urol 1994; 152:987–9. 8051779 10.1016/s0022-5347(17)32638-1

[27] Jayakumar S, Antao B, Bevington O et al Balanitis xerotica obliterans in children and its incidence under the age of 5 years. J Pediatr Urol 2012; 8:272–5. 21705275 10.1016/j.jpurol.2011.05.001

[28] Yardley IE, Cosgrove C, Lambert AW. Paediatric preputial pathology: are we circumcising enough? Ann R Coll Surg Engl 2007; 89:62–5. 17316525 10.1308/003588407X160828 PMC1963523

[29] Bunker CB, Cohen CR. Clinical and histological characteristics of lichen sclerosus in children: an updated review. Dermatol Clin 2022; 40:447–55.

[30] Kiss A, Király L, Kutasy B, Merksz M. High incidence of balanitis xerotica obliterans in boys with phimosis: prospective 10-year study. Pediatr Dermatol 2005; 22:305–8. 16060864 10.1111/j.1525-1470.2005.22404.x

[31] Dalal V, Kaur M, Rai CB et al Histopathological spectrum of lichen sclerosus et atrophicus. Indian J Dermatopathol Diagn Dermatol 2017; 4:8–13.

[32] Fistarol SK, Itin PH. Diagnosis and treatment of lichen sclerosus: an update. Am J Clin Dermatol 2013; 14:27–47. 23329078 10.1007/s40257-012-0006-4 PMC3691475

[33] Edmonds EVJ, Oyama N, Chan I et al Extracellular matrix protein 1 autoantibodies in male genital lichen sclerosus. Br J Dermatol 2011; 165:218–19. 21428972 10.1111/j.1365-2133.2011.10326.x

[34] Bunker CB . Male genital lichen sclerosus and tacrolimus. Br J Dermatol 2007; 157:1079–80. 17854373 10.1111/j.1365-2133.2007.08179.x

[35] Watchorn RE, Bunker CB. The etiopathogenesis of male genital lichen sclerosus: the foreskin, urine, and the microbiome. J Am Acad Dermatol 2019; 81:AB284.

[36] Kyriakis KP, Emmanuelides S, Terzoudi S et al Gender and age prevalence distributions of morphea en plaque and anogenital lichen sclerosus. J Eur Acad Dermatol Venereol 2007; 21:825–6. 17567317 10.1111/j.1468-3083.2006.01954.x

[37] Balakirski G, Grothaus J, Altengarten J, Ott H. Paediatric lichen sclerosus: a systematic review of 4516 cases. Br J Dermatol 2020; 182:231–3. 31260081 10.1111/bjd.18267

[38] Doiron PR, Bunker CB. Obesity-related male genital lichen sclerosus. J Eur Acad Dermatol Venereol 2017; 31:876–9. 27891728 10.1111/jdv.14035

[39] McSorley A, Nigam AK. Is routine histology necessary in circumcision? Br J Med Surg Urol 2011; 4:148–51.

[40] Alyami FA, Heidari Bateni Z, Odeh R et al Routine histopathological examination of the foreskin after circumcision for clinically suspected lichen sclerosus in children: is it a waste of resources? Can Urol Assoc J 2018; 12:E231–3. 29405913 10.5489/cuaj.4331 PMC5966935

[41] Bighetti S, Mancon S, Suardi N et al Evaluating lichen sclerosus in phimosis: insights from a multidisciplinary retrospective study. Australas J Dermatol 2025; 66:e39–42. 39812233 10.1111/ajd.14417 PMC11898142

[42] Stehr M, Bogaert G, Radmayr C et al Paediatric urology guideline. In: EAU Guidelines. Presented at the EAU Annual Congress, Milan 2023. Arnhem, The Netherlands: European Association of Urology, 2023; 10–14.

