# Correction to: Lichen sclerosus in paediatric patients with phimosis undergoing circumcision

**DOI:** 10.1093/skinhd/vzag119

**Published:** 2026-07-03

This is a correction to: Nada Aboukhatwah, Sandra Jerkovic Gulin, Christopher B Bunker, Michalis Varnavas, Georgios Kravvas, Lichen sclerosus in paediatric patients with phimosis undergoing circumcision, *Skin Health and Disease*, Volume 5, Issue 6, December 2025, Pages 426–431, https://doi.org/10.1093/skinhd/vzaf065

In the originally published version of this manuscript, the abbreviation for ‘Male Genital Lichen Sclerosus’ was incorrectly stated as ‘MGLS’. The correct abbreviation is ‘MGLSc’.

This error has been corrected.

